# Association between subjective memory complaints and health care utilisation: a three-year follow up

**DOI:** 10.1186/1471-2318-9-43

**Published:** 2009-09-23

**Authors:** Frans Boch Waldorff, Volkert Siersma, Gunhild Waldemar

**Affiliations:** 1 Section and Research Unit of General Practice, Institute of Public Health, University of Copenhagen, Denmark; 2 Memory Disorders Research Group, Department of Neurology, Rigshospitalet, Copenhagen University Hospital, Denmark

## Abstract

**Background:**

Subjective memory complaints (SMC) are common among elderly patients and little is know about the association between SMC and health care utilisation. Thus, the aim of this study was to investigate health care utilisation during a three-year follow-up among elderly patients consulting their general practitioner and reporting subjective memory complaints (SMC).

**Methods:**

This study was conducted as a prospective cohort survey in general practice with three-year follow-up. Selected health care utilisation or costs relative to SMC adjusted for potential confounders were analyzed in a two-part model where the incidence of use of a selected health care service were analyzed separately from the quantity of use for those that use the service. The former analyzed in a Poisson regression approach, the latter in a generalized linear regression model.

**Results:**

A total 758 non-nursing home residents aged 65 years and older consulted their GP in October and November 2002 and participated in the present study. The adjusted probability of nursing home placement was significantly increased in subjects with SMC relative to subjects without SMC (RR = 2.3). More generally, SMC was associated with an increase in the cost of selected health care utilisation of 60% over three years (p = 0.003).

**Conclusion:**

The data of this study indicated that in an elderly primary care population the presence of SMC increased the cost of health care utilisation by 60% over three years. Thus, inquiry into SMC may contribute to a risk profile assessment of elderly patients and may identify patients with an increased use of health care services.

## Background

In studies of older patients, the reported prevalence of subjective memory complaints (SMC) shows a huge variation with figures ranging from 10-56% [1,2]. The large variation may be explained by sample selection or by the methods applied for assessing SMC [1]. Studies have consistently associated SMC with depression [2-4], as well as personality traits [5], high age, low education and female gender [1]. A Danish study indicated that these patients rarely share their perception of SMC with their General Practitioner (GP) spontaneously [6], even though SMC may identify frail patients and inquiry into SMC may easily be implemented in a busy GP routine consultation.

In some studies, association has been found between memory complaints and cognitive impairment on testing, even after adjustment for depressive symptoms [7,8]. However, longitudinal studies assessing the value of SMC in predicting dementia or cognitive decline have shown varying results [9-16]. Thus, the nature of SMC is complex [17].

In a study from 1999 among 8775 non-institutionalized persons aged 65 or more, a single question about health strongly predicted subsequent health care utilisation after a year [18]. Other research suggests that patients with mental health conditions use general medical services at a higher rate than those without mental health conditions [19-21]. Furthermore, dementia has been associated with increased health care utilisation in several studies [22,23]. In our recent study, SMC was associated with an increased probability for nursing home placement over 4 years following the assessment [24]. However, we did not identify any other studies addressing the association between the presence of SMC and health care utilisation. Thus, the aim of the present prospective study was to investigate health care utilisation during a three-year follow-up among elderly patients with and without SMC consulting their general practitioner.

## Methods

### Study Population

All 17 practices comprising a total of 24 GPs in the central district of the municipality of Copenhagen, Denmark, participated in this study. A total of 40.865 patients were listed and 2.934 were 65 or older. Patients' aged 65 and older consulting their GP, regardless of reason for the encounter, were asked to participate in the study and received information both verbally and written. All participants signed an informed consent declaration and were not offered a refund. Patients not able to speak or read Danish, patients living in a nursing home, and patients with severe acute or terminal illness, or specialist-diagnosed patients with dementia were excluded. Non-participants were defined as those who were not excluded because of the exclusion criteria, but refused to participate. The participants were enrolled during October and November 2002.

### Outcome

End-point variables were GP related contacts, out-of-hour services, hospitalization and nursing home placement within a three-year period from enrolment, and a cumulated value of these services.

### Measurements

In brief, the examination contained:

1) A self-administered *participant questionnaire *concerning aspects of memory and sociodemographics. Information on SMC was obtained from the following item: *"How would you judge your memory?" *Theresponse categories were: "excellent", "good", "less good", "poor", or "miserable". Patients rating their memory as "less good", "poor" or "miserable" were classified as patients with SMC, while patients rating their memory as "excellent" or "good" were defined as patients without SMC.

2) A self-administered *quality of life assessment*. The patients completed the Danish Validated Version of Euro-Qol-5D. Euro-Qol-5D is a standardised instrument for use as a measure of health outcome and measures five dimensions - mobility, self-care, usual activities, pain/discomfort, and anxiety/depression - each by three levels of severity [25]. The anxiety/depression dimension was used as a proxy for depression.

3) A GP- or nurse- administered Mini Mental State Exermination (MMSE). The MMSE, a widely distributed test recommended in GP guidelines as a cognitive screening test, was completed after the completion of the GP questionnaire [26]. The MMSE score ranges from 0-30; a score lower than 24 was taken as indicative of cognitive impairment.

#### Registry data

In Denmark, much health information is collected in national registers based on a unique personal identification number allocated to each inhabitant [27]. Information concerning incident deaths, hospital contacts and GP consultations were retrieved from the central national databases by the statistical department of the Danish National Board of Health at the end of 2007. The municipality of Copenhagen provided information concerning nursing home placement at the end of 2006.

In this study the following outcomes were investigated in the three-year period from January 1^st ^2003 until 31^st ^December 2005:

1) Practice consultations (number of consultations)

2) Home visit consultations by GP (number of visits)

3) GP out-of-hours contacts (number of contacts)

4) Hospital admission (days in hospital, not as out-patient)

5) Out-patient stay (days in outpatient clinic)

6) Emergency room consultations (number of visits)

7) Nursing home placement (days in institution).

Health care utilisation was defined as the sum of the number of services or time (days) of stay over the three-year follow-up period; or a valuation based on the prices in Table 1. For those, who had died (and thereby did not use health care services during all three years), the nominal outcome was multiplied with the inverse of the proportion of the three years the subject was alive. Annualized outcomes were constructed by dividing the three-year outcomes by three.

**Table 1:** Valuation of selected health care services

**Service**	**Unit**	**Value**^**1**^	**Source**
Practice consultations	1 consultation	€ 14,39	Danish health insurance register (SSR)
Visits by GP	1 visit	€ 23,81	Danish health insurance register (SSR)
Hospital stay (not as outpatient)	1 admission day	€ 470,84	Journal of the Danish Medical Association 2005; 167 (07): 807
Outpatient stay	1 admission day	€ 187,39	The National Board of Health (drg.dk)
Out-of-hours contacts	1 contact	€ 14,66	Danish health insurance register (SSR)
Emergency	1 visit	€ 105,74	The National Board of Health (drg.dk)
Nursing home	1 admission day	€ 127,80	Journal of the Danish Medical Association 2005; 167 (07): 807

^1^2004 prices in DKK converted to EUR using the july 1st 2004 spot rate DKK743.35 = EUR100 (source: Danish national bank )

### Statistical analysis

Differences in characteristics and health care utilisation between participants with and without SMC were tested by chi-squared tests. A total cost for the health care utilisation was calculated using the valuation in Table 1; the difference in this cost between participants with and without SMC was analyzed with a Kruskal-Wallis non-parametric test. Differences in total cost between subgroups of the participants were tested by the F-test of the regression parameter(s) corresponding to the characteristic classifying the subgroups in a linear regression on total cost, additionally adjusted for SMC. These tests evaluated the effect of the characteristic on the total cost beyond the part of the effect that was mediated by SMC.

Multivariate analysis of health care utilisation followed a two-part model where the incidence of use (ever used) of a selected health care service was analyzed separately from the quantity of use for those that use the service [28]. The incidence was analyzed in a Poisson regression approach [29] so that the regression parameters were equivalent to the log of the relative risk (RR) of using the service ever in the study period. For the participants that use the service (or have cost>0) the quantity of use was analyzed in a generalized linear model using a Gamma distribution and a logarithmic link function; the parameters from this model were interpreted as the log of a (multiplicative) factor how much more the service was used compared to a baseline class. A combined (multiplicative) effect of having SMC compared to not having SMC was straightforwardly calculated by multiplying the RR from the first part and the factor from the second part. Statistical significance was assessed at a 5% level. We adjusted for multiple testing by the method of Benjamini-Hochberg in the final multivariate analysis [30].

### Ethics

The Scientific Ethical Committee for Copenhagen and Frederiksberg Municipalities evaluated the project. The Danish Data Protection Agency, the Danish College of General Practitioners Study Committee as well as The National Board of Health approved the project.

## Results

**Figure 1:**
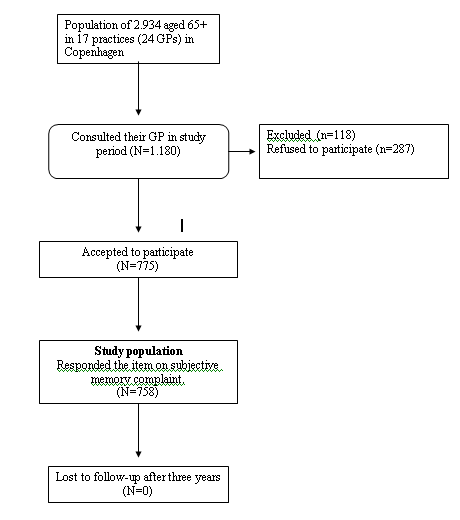
**Flowchart of Study population**.

The final cohort consisted of 775 non-nursing home residents of which 758 filled out the SMC item. Figure 1 shows the trial flow. The average age of participants at baseline was 74.8 of whom 38.6% were males; average MMSE was 28.2 (range: 16-30). According to our definition 177 (23%) had SMC at baseline. Non-participants were more likely to be males (OR = 1.4) and were, according to the GP, less likely to complain about memory problems, (OR = 1.8). All participants were followed up until the end of 2005 and none were lost to follow-up.

During the study period 88 (11.6%) died and 50 (6.6%) were admitted to nursing homes. A total of 701 (92.5%) had at least one GP consultation and 432 (60.0%) have at least one hospital admission during the study period. Furthermore, SMC is not seen to correlate with MMSE (Table 2). Valuations of selected health care services are shown in Table 1.

**Table 2:** Baseline characteristics and health care utilisation of the study participants (n = 758) by Subjective Memory Complaints (SMC)

		**SMC**					
		**No** **(n = 581)**		**Yes** **(n = 177)**			
		**n**	**%**	**n**	**%**	**Sign.**	**Missing**
Death	no	517	89,0	153	86,4		
	yes	64	11,0	24	13,6		
MMSE	≥ 24	555	95,5	165	93,2		
	< 24	26	4,5	12	6,8		
Age	60 - 74	318	54,7	86	48,6		
	75 - 84	207	35,6	68	38,4		
	85+	56	9,6	23	13,0		
Sex	male	233	40,1	61	34,5		
	female	348	59,9	116	65,5		
Living without partner	no	240	41,4	60	34,3		3
	yes	340	58,6	115	65,7		
Education	< 8 years	226	38,9	69	39,0		
	> 8 years	355	61,1	108	61,0		
Home care	no	473	81,7	126	72,0	***	4
	yes	106	18,3	49	28,0		
Mobility^1^	no problems	384	67,3	90	52,0	***	14
	some problems	187	32,7	83	48,0		
Self-care^1^	no problems	539	94,7	158	90,8		15
	some problems	30	5,3	16	9,2		
Usual activities^1^	no problems	412	72,5	84	48,6		
	some problems	145	25,5	84	48,6	***	17
	severe problems	11	1,9	5	2,9		
Pain/discomfort^1^	no	216	38,4	45	25,9		
	moderate	323	57,4	111	63,8	***	21
	extreme	24	4,3	18	10,3		
Anxiety/depression^1^	no	442	77,8	98	57,0		
	moderate	115	20,2	71	41,3	***	18
	extreme	11	1,9	3	1,7		
*Health Care Utilization*							
Practice consultations^2^	no	41	7,1	16	9,0		
	yes	540	92,9	161	91,0		
Visits by GP^2^	no	422	72,6	117	66,1		
	yes	159	27,4	60	33,9		
Hospital stay (no outpatient)^2^	no	259	44,6	67	37,9		
	yes	322	55,4	110	62,1		
Outpatient stay^2^	no	165	28,4	34	19,2	*	
	yes	416	71,6	143	80,8		
Out-of-hours contact^2^	no	548	94,3	170	96,0		
	yes	33	5,7	7	4,0		
Emergency^2^	no	345	59,4	82	46,3	**	
	yes	236	40,6	95	53,7		
Nursing home^2^	no	554	95,4	154	87,0	***	
	yes	27	4,6	23	13,0		

* significant at 5% level ** significant at 1% level *** significant at 0.1% level, ^1^based on Euro-Qol-5D, for mobility and self-care the third category did not appear because of the method of data collection, ^2^incidence in the period 2003-2005.

Annualized cost (in EUR) of health care utilisation by SMC and participant characteristics is shown in Table 3. Lower MMSE scores, increased age, lower education, home care and lower physical activity increased the cost of health care utilisation. The differences in health care utilisation and costs attributable to SMC, i.e. adjusted for the characteristics listed in Table 3, are shown in Table 4. The presence of SMC significantly increased the probability of nursing home placement (RR = 2.3). More generally, SMC was significantly associated with an increase in health care costs for the combined selected services over the three years of follow-up by 60%. When the cost of nursing home admission is omitted from the total cost analysis, SMC is associated only with a non-significant 23% increase

**Table 3:** Annualised cost (EUR) of health care utilisation by Subjective Memory Complaints (SMC) and participant characteristics

		**SMC**						
		**No (n = 581)**			**Yes (n = 177)**			
		**Median**	**IQR**		**Median**	**IQR**		**Sign.**^**1**^
Total cost (EUR)		838	192	3389	1577	597	9894	***^2^
MMSE	≥24	831	192	3209	1457	548	7620	**
	< 24	4572	183	13033	9888	2597	22082	
Age	60 - 74	566	178	2170	993	274	1659	
	75 - 84	1143	226	4438	3321	1076	14743	***
	85+	3277	494	24366	14609	1713	27545	
Sex	male	1036	202	3637	1190	322	3302	
	female	794	187	3360	2187	733	13738	
Living without partner	no	815	154	2601	1225	541	4135	
	yes	842	219	3931	2125	695	14770	
Education	< 8 years	944	185	4151	2998	528	17280	*
	> 8 years	831	197	2750	1383	612	6291	
Home care	no	660	182	2551	1069	307	2998	***
	yes	2642	682	9065	14609	3329	23801	
Mobility	no problems	613	163	2327	1177	280	4985	***
	some problems	1778	313	8509	2883	958	14770	
Self-care	no problems	832	192	3294	1431	548	6755	**
	some problems	2027	288	8896	13416	2788	20493	
Usual activities	no problems	605	163	2380	1194	301	4160	
	some problems	1891	433	6497	2556	767	13791	
	severe problems	1531	178	14788	3615	3248	23152	***
Pain/discomfort	no	594	133	2227	1811	695	14609	
	moderate	1063	222	4266	1510	543	6161	
	extreme	1877	324	6137	2669	1050	12867	
Anxiety/depression	no	794	187	3182	1494	548	8905	
	moderate	1036	226	3595	1577	682	9894	
	extreme	13606	887	17944	12512	7620	30547	

*significant at 5% level ** significant at 1% level *** significant at 0.1% level

^1^Significance of the regression parameter of the corresponding participant characteristic in a linear regression on total cost, adjusted for SMC

^2^Wilcoxon non-parametric test

**Table 4:** Selected health care utilisation and costs in subjects with Subjective Memory Complaints (SMC) relative to patients without SMC^1^

	**The RR of any use of the corresponding service at all**				**Factor how much more people with SMC use the service**				**Combined effect**
**Service**	**RR**	**95% CI**		**p-value**^**2**^	**Factor**	**95% CI**		**p-value**^**2**^	
*GP contacts*									
Practice consultations	0,976	0,922	1,032	0,3924	0,988	0,866	1,126	0,8559	0,964
Visits by GP	1,116	0,876	1,421	0,3863	0,967	0,717	1,304	0,8255	1,079
GP contacts (cost)	0,970	0,920	1,023	0,2610	1,001	0,883	1,135	0,9830	0,972
*Hospital stay*									
Hospital stay (not as outpatient, days)	1,052	0,911	1,216	0,4953	1,189	0,895	1,582	0,2282	1,252
Outpatient stay (days)	1,111	1,010	1,221	0,0344	1,082	0,875	1,338	0,4663	1,202
Hospital stay (cost)	1,061	0,978	1,151	0,1611	1,178	0,920	1,509	0,1896	1,250
*Out-of-hours services*									
Out-of-hours GP contacts	0,575	0,237	1,398	0,1686	1,437	1,092	1,891	0,0116	0,827
Emergency (visits)	1,209	1,007	1,452	0,0512	1,073	0,916	1,256	0,3828	1,297
Out-of-hours services (cost)	1,121	0,939	1,340	0,2183	1,172	0,977	1,405	0,0858	1,314
*Nursing home*									
Nursing home (days)	2,296	1,357	3,886	0,0075	0,922	0,686	1,238	0,5900	2,117
Nursing home (cost)	2,296	1,357	3,886	0,0075	0,922	0,686	1,238	0,5900	2,117
									
**The above combined (cost)**	**0,990**	**0,961**	**1,020**	**0,5070**	**1,615**	**1,234**	**2,114**	**0,0003**	**1,599**

^1^All analyses adjusted for the participant characteristics presented in Table 3

^2^Due to multiple testing the level of significance is set to 0.0081

## Discussion

To our knowledge, this is the first study to demonstrate that in elderly patients SMC was attributable to an increase in cost by 60% over three years for selected health care services. Specifically, SMC increased the probability of nursing home placement. Much of the excess cost in the SMC group seems to be explained by the higher frequency of nursing home admission.

SMC is a commonly reported symptom in the elderly [1,2]. In this study we adjusted for commonly known confounders e.g. depression and cognitive performance, and the result indicated that the increase in health care utilisation attributed to SMC was substantial. The tendency, that nursing home placement was increased has been reported previously using data from this study. The increased health care utilisation may not solely be explained by nursing home admission. Tendencies of increased use of out-patient clinic admissions and out-of-hour services can be observed. In contrast, the use of GP daytime consultations and acute hospital admittance were not increased.

The reported effect of SMC was beyond various other potential confounders. It is well-known that the presence of dementia in general is associated with an increased health care utilisation [31]. This is in accordance with this study, where our item indicating that significant cognitive impairment (defined as MMSE less than 24) was an independent predictor for nursing home placement. Also, depression in old age has also consistently been associated with an increased health care costs, even after controlling for chronic medical co-morbidity [32]. Our study found that age, but not depressive symptoms were associated with an increased health care utilisation. Furthermore, low education increased health care utilisation. The absence of correlation between SMC and cognitive functioning (MMSE) stresses their different psychometric properties. We assume that SMC measures a global functioning in elderly patients. In Table 2 it can be seen that there is no notable difference in mortality between the subjects with and without SMC. Hence, the difference in health care utilisation and costs cannot be attributed to the high end-of-life utilisation and costs that are generally observed.

The mechanism by which SMC leads to increased health care utilisation is, in our view, not a direct causative relation. However, we see a statistical association between SMC and health care utilisation as residual confounding, i.e. there are certain factors - possibly unknown or immeasurable - beyond the covariates that are used in the analyses, that cause the subject to have memory complaints *and *cause increased health care utilisation.

The sampling of the participants reflects the population in which the GP has an opportunity to ask questions about SMC. Thus, we deliberately designed the study to include a patient sample, which reflects daily clinical practice. The nation-wide databases used in order to evaluate our main outcomes are regarded as highly valid. Thus, we believe that our findings are valid.

The statistical analysis was done in a two-part model according to recommendations [28]. Data tend to conform to the analytic assumptions for these models, and the models can be used to gain insight in the process of health care utilisation. The decision to have any use at all of a certain service is most likely made by the person and so is related primarily to personal characteristics, while the cost and frequency per user may be more related to characteristics of the health care system.

Several limitations must be addressed. This study had some selection biases at baseline, which may decrease generalizability. Only elderly persons who consulted their GP for whatever reason were included, and they may be more vulnerable than elderly persons in the general population. We did not have access to databases regarding medication, which would have been relevant to evaluate. Likewise, we did not obtain information about medical diagnosis in the participants, as diagnostic criteria are not systematically implemented in general practice in Denmark, and we wanted the study to reflect current standards. Participants who had already been diagnosed with dementia by a specialist were excluded from the study, which is reflected by the high average MMSE in our study population. A MMSE score less than 24 has been widely used as an indication of the presence of cognitive impairment in population based studies [33]. However, epidemiological research has shown that MMSE scores are affected by age, education, and cultural background [33] and MMSE is not sufficient to diagnose dementia. In our study we used the depression item in the Euro-Qol-5D to identify patients with self reported anxiety and depression. These patients may not fulfill international criteria for anxiety and depression. However, this item may serve as indicator for affective symptoms.

There is a lack of consensus concerning the assessment of SMC. Some studies have assessed the presence of SMC by a single item, others by several items. In this study, a single item was used to assess SMC. This item did not allow us to know whether the patient was calibrating the response by comparing to former functioning or to the functioning of others. Notably, our SMC item did not distinguish between short-term and long-term memory loss. We recommend that future studies give more attention to this specific aspect and also include informant reports on memory.

## Conclusion

The data suggest that in an elderly primary care population SMC is associated with an increased health care utilisation by 60%, primarily because of increased nursing home placement. Therefore, the result of this study indicates that GPs may identify elderly patients with an increased probability of subsequent health care utilisation by routinely inquiring about memory problems.

## Competing interests

The authors declare that they have no competing interests.

## Authors' contributions

FBW conceived the study concept, design, funding, data analysis, interpretation, and wrote the first draft of the manuscript. VS participated in the data analysis, interpretation, and manuscript preparation. GW participated in the study concept, design, data analysis, interpretation, and manuscript preparation. All authors read and approved the final manuscript.

## Pre-publication history

The pre-publication history for this paper can be accessed here:


